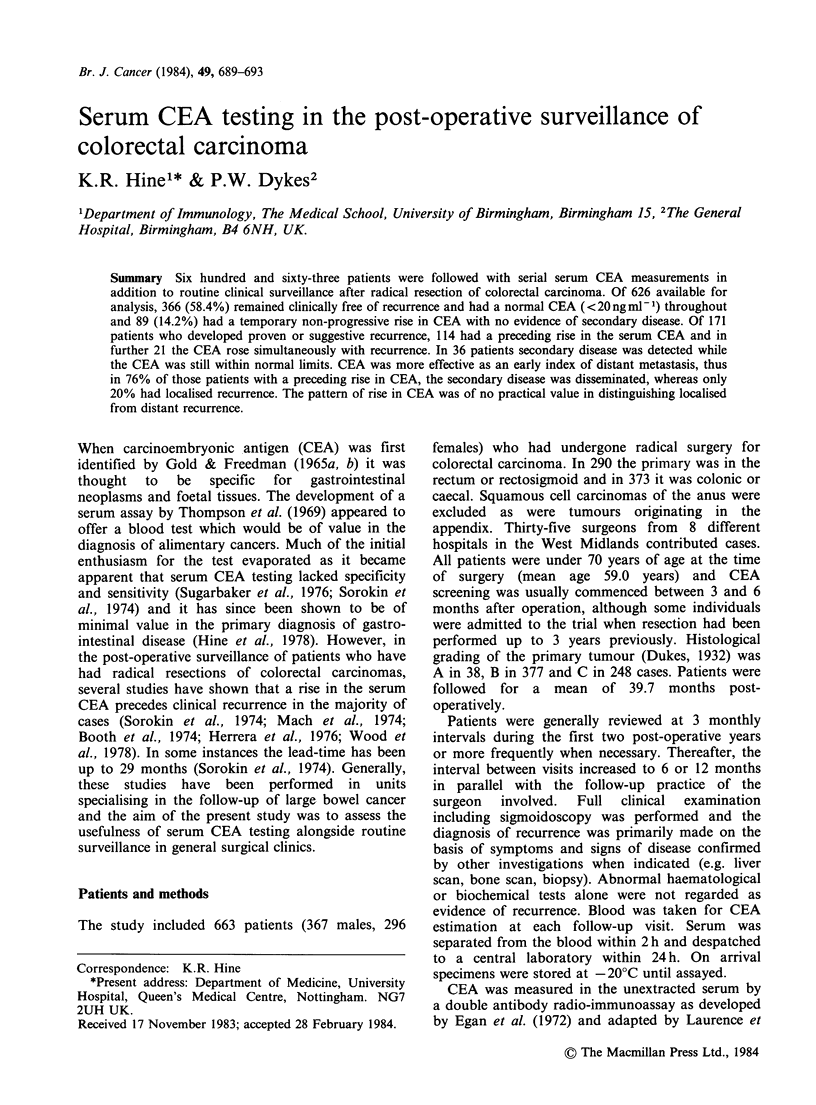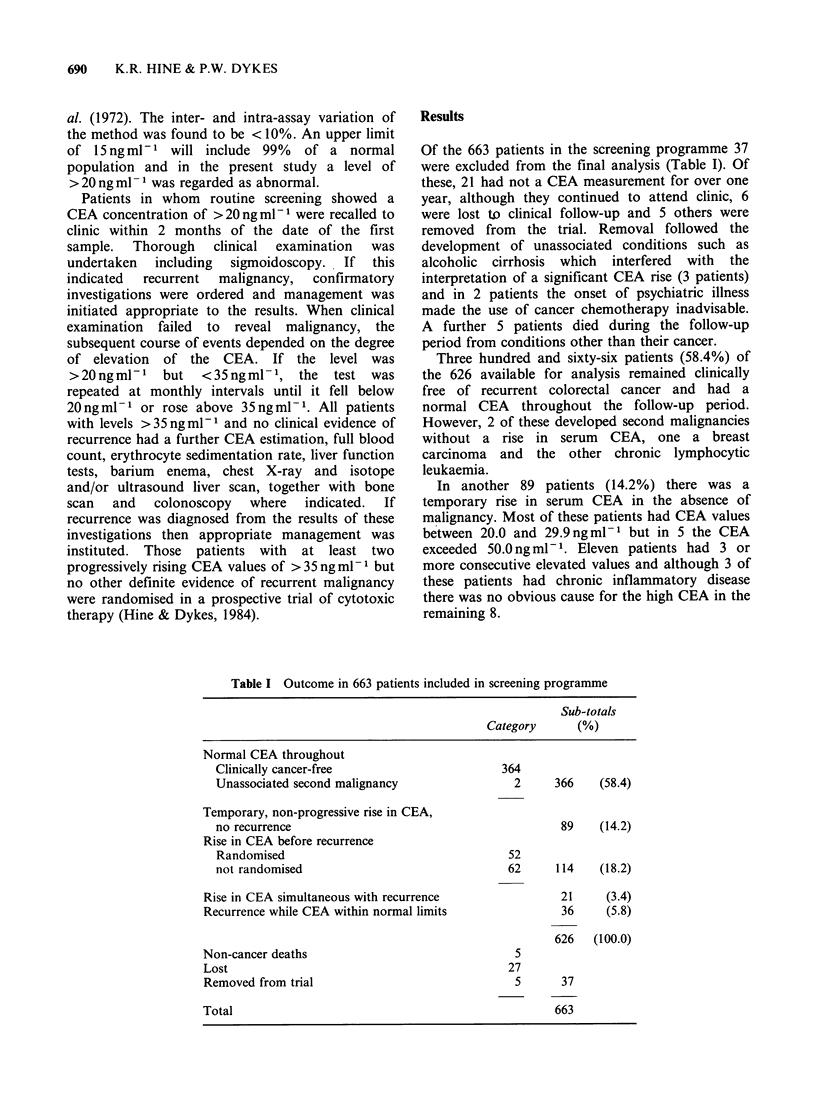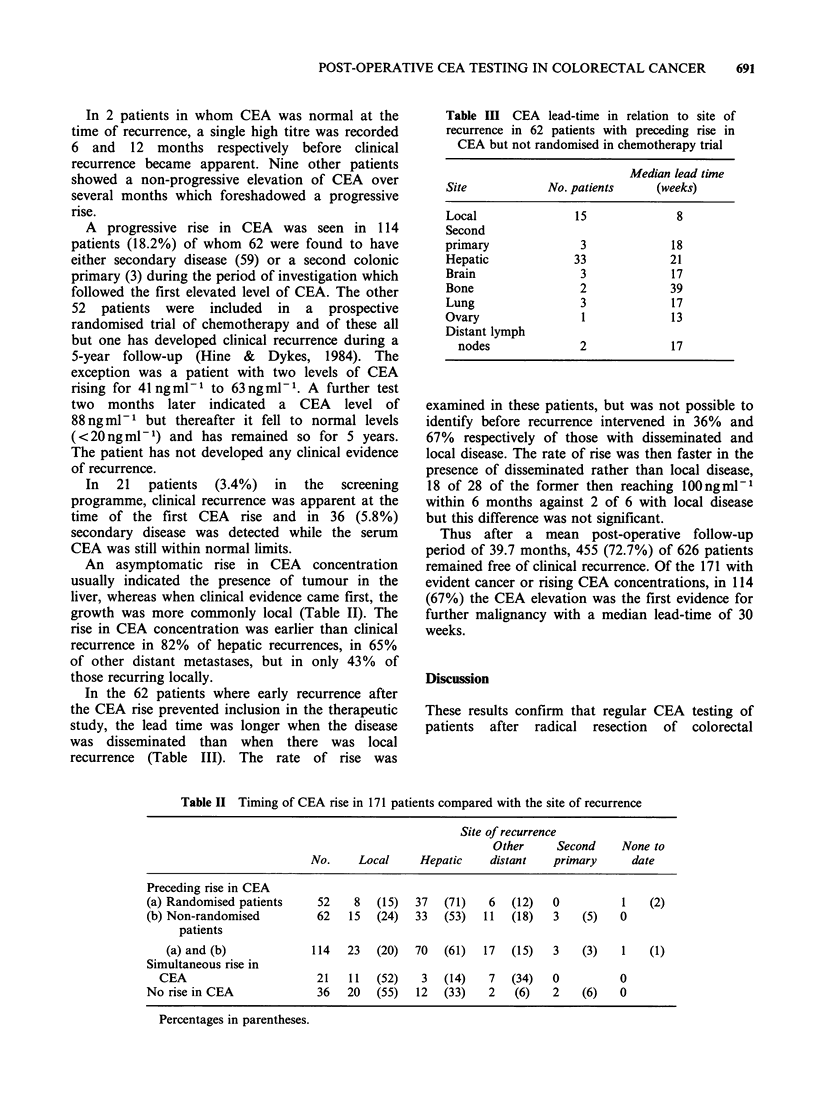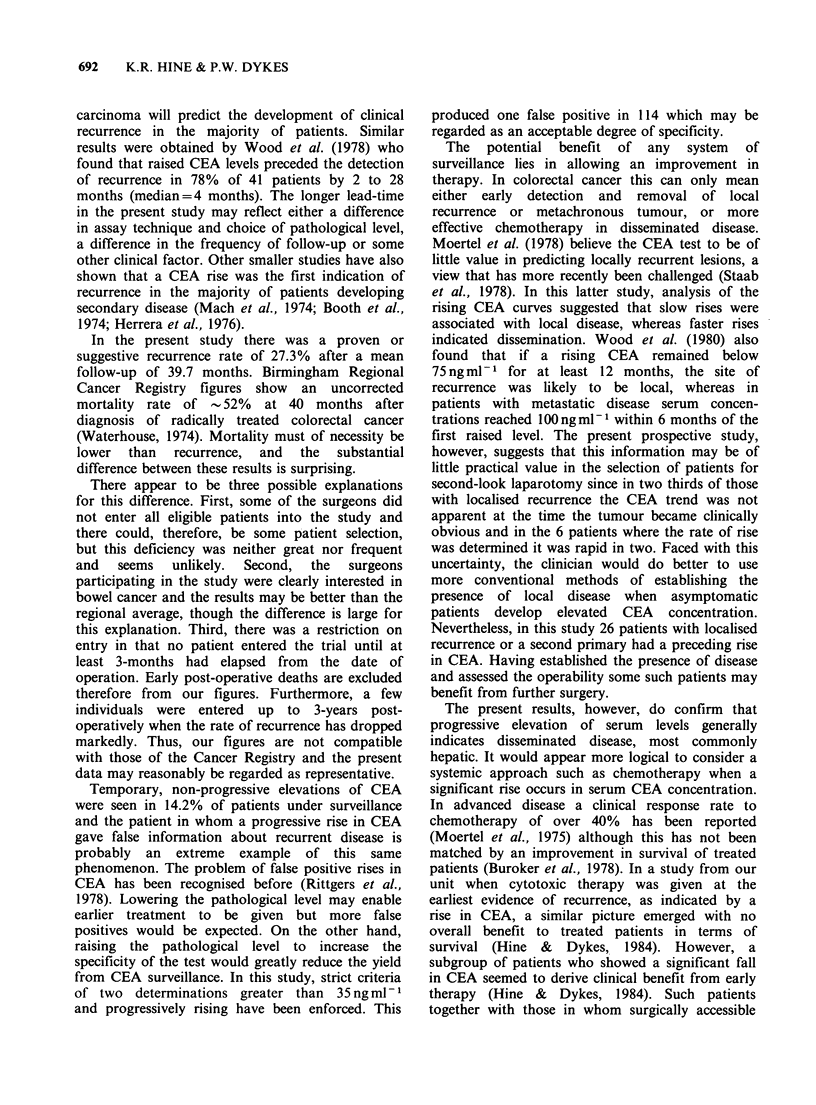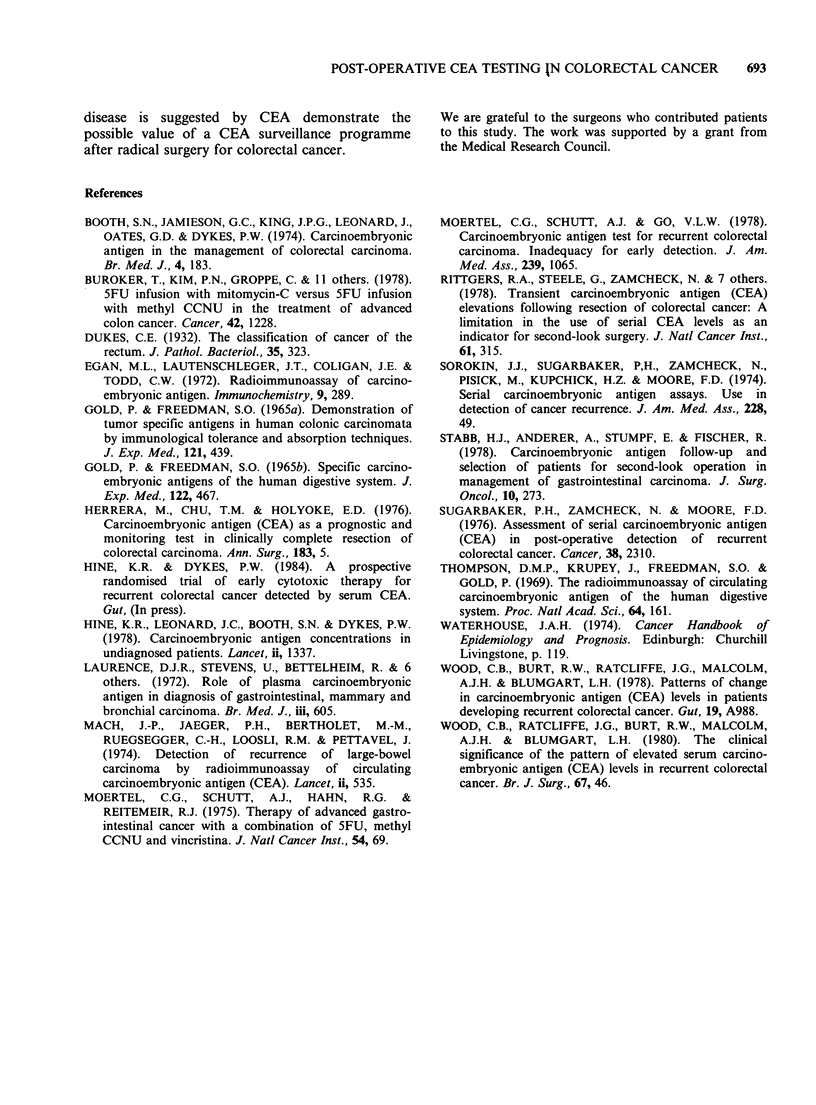# Serum CEA testing in the post-operative surveillance of colorectal carcinoma.

**DOI:** 10.1038/bjc.1984.109

**Published:** 1984-06

**Authors:** K. R. Hine, P. W. Dykes

## Abstract

Six hundred and sixty-three patients were followed with serial serum CEA measurements in addition to routine clinical surveillance after radical resection of colorectal carcinoma. Of 626 available for analysis, 366 (58.4%) remained clinically free of recurrence and had a normal CEA (less than 20 ng ml-1) throughout and 89 (14.2%) had a temporary non-progressive rise in CEA with no evidence of secondary disease. Of 171 patients who developed proven or suggestive recurrence, 114 had a preceding rise in the serum CEA and in further 21 the CEA rose simultaneously with recurrence. In 36 patients secondary disease was detected while the CEA was still within normal limits. CEA was more effective as an early index of distant metastasis, thus in 76% of those patients with a preceding rise in CEA, the secondary disease was disseminated, whereas only 20% had localised recurrence. The pattern of rise in CEA was of no practical value in distinguishing localised from distant recurrence.


*Br. J. Cancer* (1984), **49**, 689–693

### Serum CEA testing in the post-operative surveillance of colorectal carcinoma

K.R. Hine[1]* & P.W. Dykes[2]

[1]*Department of Immunology, The Medical School, University of Birmingham, Birmingham 15,* [2]*The General Hospital, Birmingham, B4 6NH, UK.*

**Summary** Six hundred and sixty-three patients were followed with serial serum CEA measurements in addition to routine clinical surveillance after radical resection of colorectal carcinoma. Of 626 available for analysis, 366 (58.4%) remained clinically free of recurrence and had a normal CEA ($<20\,\text{ng ml}^{-1}$) throughout and 89 (14.2%) had a temporary non-progressive rise in CEA with no evidence of secondary disease. Of 171 patients who developed proven or suggestive recurrence, 114 had a preceding rise in the serum CEA and in further 21 the CEA rose simultaneously with recurrence. In 36 patients secondary disease was detected while the CEA was still within normal limits. CEA was more effective as an early index of distant metastasis, thus in 76% of those patients with a preceding rise in CEA, the secondary disease was disseminated, whereas only 20% had localised recurrence. The pattern of rise in CEA was of no practical value in distinguishing localised from distant recurrence.

When carcinoembryonic antigen (CEA) was first identified by Gold & Freedman (1965*a*, *b*) it was thought to be specific for gastrointestinal neoplasms and foetal tissues. The development of a serum assay by Thompson *et al.* (1969) appeared to offer a blood test which would be of value in the diagnosis of alimentary cancers. Much of the initial enthusiasm for the test evaporated as it became apparent that serum CEA testing lacked specificity and sensitivity (Sugarbaker *et al.*, 1976; Sorokin *et al.*, 1974) and it has since been shown to be of minimal value in the primary diagnosis of gastrointestinal disease (Hine *et al.*, 1978). However, in the post-operative surveillance of patients who have had radical resections of colorectal carcinomas, several studies have shown that a rise in the serum CEA precedes clinical recurrence in the majority of cases (Sorokin *et al.*, 1974; Mach *et al.*, 1974; Booth *et al.*, 1974; Herrera *et al.*, 1976; Wood *et al.*, 1978). In some instances the lead-time has been up to 29 months (Sorokin *et al.*, 1974). Generally, these studies have been performed in units specialising in the follow-up of large bowel cancer and the aim of the present study was to assess the usefulness of serum CEA testing alongside routine surveillance in general surgical clinics.

### Patients and methods

The study included 663 patients (367 males, 296 females) who had undergone radical surgery for colorectal carcinoma. In 290 the primary was in the rectum or rectosigmoid and in 373 it was colonic or caecal. Squamous cell carcinomas of the anus were excluded as were tumours originating in the appendix. Thirty-five surgeons from 8 different hospitals in the West Midlands contributed cases. All patients were under 70 years of age at the time of surgery (mean age 59.0 years) and CEA screening was usually commenced between 3 and 6 months after operation, although some individuals were admitted to the trial when resection had been performed up to 3 years previously. Histological grading of the primary tumour (Dukes, 1932) was A in 38, B in 377 and C in 248 cases. Patients were followed for a mean of 39.7 months post-operatively.

Patients were generally reviewed at 3 monthly intervals during the first two post-operative years or more frequently when necessary. Thereafter, the interval between visits increased to 6 or 12 months in parallel with the follow-up practice of the surgeon involved. Full clinical examination including sigmoidoscopy was performed and the diagnosis of recurrence was primarily made on the basis of symptoms and signs of disease confirmed by other investigations when indicated (e.g. liver scan, bone scan, biopsy). Abnormal haematological or biochemical tests alone were not regarded as evidence of recurrence. Blood was taken for CEA estimation at each follow-up visit. Serum was separated from the blood within 2 h and despatched to a central laboratory within 24 h. On arrival specimens were stored at −20°C until assayed.

CEA was measured in the unextracted serum by a double antibody radio-immunoassay as developed by Egan *et al.* (1972) and adapted by Laurence *et*

Correspondence: K.R. Hine
*Present address: Department of Medicine, University Hospital, Queen's Medical Centre, Nottingham. NG7 2UH UK.
Received 17 November 1983; accepted 28 February 1984.

© The Macmillan Press Ltd., 1984

*al.* (1972). The inter- and intra-assay variation of the method was found to be <10%. An upper limit of $15\,ng\,ml^{-1}$ will include 99% of a normal population and in the present study a level of $>20\,ng\,ml^{-1}$ was regarded as abnormal.

Patients in whom routine screening showed a CEA concentration of $>20\,ng\,ml^{-1}$ were recalled to clinic within 2 months of the date of the first sample. Thorough clinical examination was undertaken including sigmoidoscopy. If this indicated recurrent malignancy, confirmatory investigations were ordered and management was initiated appropriate to the results. When clinical examination failed to reveal malignancy, the subsequent course of events depended on the degree of elevation of the CEA. If the level was $>20\,ng\,ml^{-1}$ but $<35\,ng\,ml^{-1}$, the test was repeated at monthly intervals until it fell below $20\,ng\,ml^{-1}$ or rose above $35\,ng\,ml^{-1}$. All patients with levels $>35\,ng\,ml^{-1}$ and no clinical evidence of recurrence had a further CEA estimation, full blood count, erythrocyte sedimentation rate, liver function tests, barium enema, chest X-ray and isotope and/or ultrasound liver scan, together with bone scan and colonoscopy where indicated. If recurrence was diagnosed from the results of these investigations then appropriate management was instituted. Those patients with at least two progressively rising CEA values of $>35\,ng\,ml^{-1}$ but no other definite evidence of recurrent malignancy were randomised in a prospective trial of cytotoxic therapy (Hine & Dykes, 1984).

### Results

Of the 663 patients in the screening programme 37 were excluded from the final analysis (Table I). Of these, 21 had not a CEA measurement for over one year, although they continued to attend clinic, 6 were lost to clinical follow-up and 5 others were removed from the trial. Removal followed the development of unassociated conditions such as alcoholic cirrhosis which interfered with the interpretation of a significant CEA rise (3 patients) and in 2 patients the onset of psychiatric illness made the use of cancer chemotherapy inadvisable. A further 5 patients died during the follow-up period from conditions other than their cancer.

Three hundred and sixty-six patients (58.4%) of the 626 available for analysis remained clinically free of recurrent colorectal cancer and had a normal CEA throughout the follow-up period. However, 2 of these developed second malignancies without a rise in serum CEA, one a breast carcinoma and the other chronic lymphocytic leukaemia.

In another 89 patients (14.2%) there was a temporary rise in serum CEA in the absence of malignancy. Most of these patients had CEA values between 20.0 and $29.9\,ng\,ml^{-1}$ but in 5 the CEA exceeded $50.0\,ng\,ml^{-1}$. Eleven patients had 3 or more consecutive elevated values and although 3 of these patients had chronic inflammatory disease there was no obvious cause for the high CEA in the remaining 8.

**Table I** Outcome in 663 patients included in screening programme

| | *Category* | *Sub-totals* | (%) |
|---|---|---|---|
| Normal CEA throughout | | | |
| Clinically cancer-free | 364 | | |
| Unassociated second malignancy | 2 | 366 | (58.4) |
| Temporary, non-progressive rise in CEA, no recurrence | | 89 | (14.2) |
| Rise in CEA before recurrence | | | |
| Randomised | 52 | | |
| not randomised | 62 | 114 | (18.2) |
| Rise in CEA simultaneous with recurrence | | 21 | (3.4) |
| Recurrence while CEA within normal limits | | 36 | (5.8) |
| | | 626 | (100.0) |
| Non-cancer deaths | 5 | | |
| Lost | 27 | | |
| Removed from trial | 5 | 37 | |
| Total | | 663 | |

In 2 patients in whom CEA was normal at the time of recurrence, a single high titre was recorded 6 and 12 months respectively before clinical recurrence became apparent. Nine other patients showed a non-progressive elevation of CEA over several months which foreshadowed a progressive rise.

A progressive rise in CEA was seen in 114 patients (18.2%) of whom 62 were found to have either secondary disease (59) or a second colonic primary (3) during the period of investigation which followed the first elevated level of CEA. The other 52 patients were included in a prospective randomised trial of chemotherapy and of these all but one has developed clinical recurrence during a 5-year follow-up (Hine & Dykes, 1984). The exception was a patient with two levels of CEA rising for $41 ng ml^{-1}$ to $63 ng ml^{-1}$. A further test two months later indicated a CEA level of $88 ng ml^{-1}$ but thereafter it fell to normal levels ($<20 ng ml^{-1}$) and has remained so for 5 years. The patient has not developed any clinical evidence of recurrence.

In 21 patients (3.4%) in the screening programme, clinical recurrence was apparent at the time of the first CEA rise and in 36 (5.8%) secondary disease was detected while the serum CEA was still within normal limits.

An asymptomatic rise in CEA concentration usually indicated the presence of tumour in the liver, whereas when clinical evidence came first, the growth was more commonly local (Table II). The rise in CEA concentration was earlier than clinical recurrence in 82% of hepatic recurrences, in 65% of other distant metastases, but in only 43% of those recurring locally.

In the 62 patients where early recurrence after the CEA rise prevented inclusion in the therapeutic study, the lead time was longer when the disease was disseminated than when there was local recurrence (Table III). The rate of rise was examined in these patients, but was not possible to identify before recurrence intervened in 36% and 67% respectively of those with disseminated and local disease. The rate of rise was then faster in the presence of disseminated rather than local disease, 18 of 28 of the former then reaching $100 ng ml^{-1}$ within 6 months against 2 of 6 with local disease but this difference was not significant.

**Table III** CEA lead-time in relation to site of recurrence in 62 patients with preceding rise in CEA but not randomised in chemotherapy trial

| *Site* | *No. patients* | *Median lead time (weeks)* |
|---|---|---|
| Local | 15 | 8 |
| Second primary | 3 | 18 |
| Hepatic | 33 | 21 |
| Brain | 3 | 17 |
| Bone | 2 | 39 |
| Lung | 3 | 17 |
| Ovary | 1 | 13 |
| Distant lymph nodes | 2 | 17 |

Thus after a mean post-operative follow-up period of 39.7 months, 455 (72.7%) of 626 patients remained free of clinical recurrence. Of the 171 with evident cancer or rising CEA concentrations, in 114 (67%) the CEA elevation was the first evidence for further malignancy with a median lead-time of 30 weeks.

### Discussion

These results confirm that regular CEA testing of patients after radical resection of colorectal

**Table II** Timing of CEA rise in 171 patients compared with the site of recurrence

| | *No.* | *Local* | | *Hepatic* | | *Other distant* | | *Second primary* | | *None to date* | |
|---|---|---|---|---|---|---|---|---|---|---|---|
| Preceding rise in CEA | | | | | | | | | | | |
| (a) Randomised patients | 52 | 8 | (15) | 37 | (71) | 6 | (12) | 0 | | 1 | (2) |
| (b) Non-randomised patients | 62 | 15 | (24) | 33 | (53) | 11 | (18) | 3 | (5) | 0 | |
| (a) and (b) | 114 | 23 | (20) | 70 | (61) | 17 | (15) | 3 | (3) | 1 | (1) |
| Simultaneous rise in CEA | 21 | 11 | (52) | 3 | (14) | 7 | (34) | 0 | | 0 | |
| No rise in CEA | 36 | 20 | (55) | 12 | (33) | 2 | (6) | 2 | (6) | 0 | |

Percentages in parentheses.

carcinoma will predict the development of clinical recurrence in the majority of patients. Similar results were obtained by Wood *et al.* (1978) who found that raised CEA levels preceded the detection of recurrence in 78% of 41 patients by 2 to 28 months (median = 4 months). The longer lead-time in the present study may reflect either a difference in assay technique and choice of pathological level, a difference in the frequency of follow-up or some other clinical factor. Other smaller studies have also shown that a CEA rise was the first indication of recurrence in the majority of patients developing secondary disease (Mach *et al.*, 1974; Booth *et al.*, 1974; Herrera *et al.*, 1976).

In the present study there was a proven or suggestive recurrence rate of 27.3% after a mean follow-up of 39.7 months. Birmingham Regional Cancer Registry figures show an uncorrected mortality rate of ~52% at 40 months after diagnosis of radically treated colorectal cancer (Waterhouse, 1974). Mortality must of necessity be lower than recurrence, and the substantial difference between these results is surprising.

There appear to be three possible explanations for this difference. First, some of the surgeons did not enter all eligible patients into the study and there could, therefore, be some patient selection, but this deficiency was neither great nor frequent and seems unlikely. Second, the surgeons participating in the study were clearly interested in bowel cancer and the results may be better than the regional average, though the difference is large for this explanation. Third, there was a restriction on entry in that no patient entered the trial until at least 3-months had elapsed from the date of operation. Early post-operative deaths are excluded therefore from our figures. Furthermore, a few individuals were entered up to 3-years post-operatively when the rate of recurrence has dropped markedly. Thus, our figures are not compatible with those of the Cancer Registry and the present data may reasonably be regarded as representative.

Temporary, non-progressive elevations of CEA were seen in 14.2% of patients under surveillance and the patient in whom a progressive rise in CEA gave false information about recurrent disease is probably an extreme example of this same phenomenon. The problem of false positive rises in CEA has been recognised before (Rittgers *et al.*, 1978). Lowering the pathological level may enable earlier treatment to be given but more false positives would be expected. On the other hand, raising the pathological level to increase the specificity of the test would greatly reduce the yield from CEA surveillance. In this study, strict criteria of two determinations greater than 35 ng ml$^{-1}$ and progressively rising have been enforced. This produced one false positive in 114 which may be regarded as an acceptable degree of specificity.

The potential benefit of any system of surveillance lies in allowing an improvement in therapy. In colorectal cancer this can only mean either early detection and removal of local recurrence or metachronous tumour, or more effective chemotherapy in disseminated disease. Moertel *et al.* (1978) believe the CEA test to be of little value in predicting locally recurrent lesions, a view that has more recently been challenged (Staab *et al.*, 1978). In this latter study, analysis of the rising CEA curves suggested that slow rises were associated with local disease, whereas faster rises indicated dissemination. Wood *et al.* (1980) also found that if a rising CEA remained below 75 ng ml$^{-1}$ for at least 12 months, the site of recurrence was likely to be local, whereas in patients with metastatic disease serum concentrations reached 100 ng ml$^{-1}$ within 6 months of the first raised level. The present prospective study, however, suggests that this information may be of little practical value in the selection of patients for second-look laparotomy since in two thirds of those with localised recurrence the CEA trend was not apparent at the time the tumour became clinically obvious and in the 6 patients where the rate of rise was determined it was rapid in two. Faced with this uncertainty, the clinician would do better to use more conventional methods of establishing the presence of local disease when asymptomatic patients develop elevated CEA concentration. Nevertheless, in this study 26 patients with localised recurrence or a second primary had a preceding rise in CEA. Having established the presence of disease and assessed the operability some such patients may benefit from further surgery.

The present results, however, do confirm that progressive elevation of serum levels generally indicates disseminated disease, most commonly hepatic. It would appear more logical to consider a systemic approach such as chemotherapy when a significant rise occurs in serum CEA concentration. In advanced disease a clinical response rate to chemotherapy of over 40% has been reported (Moertel *et al.*, 1975) although this has not been matched by an improvement in survival of treated patients (Buroker *et al.*, 1978). In a study from our unit when cytotoxic therapy was given at the earliest evidence of recurrence, as indicated by a rise in CEA, a similar picture emerged with no overall benefit to treated patients in terms of survival (Hine & Dykes, 1984). However, a subgroup of patients who showed a significant fall in CEA seemed to derive clinical benefit from early therapy (Hine & Dykes, 1984). Such patients together with those in whom surgically accessible disease is suggested by CEA demonstrate the possible value of a CEA surveillance programme after radical surgery for colorectal cancer.

We are grateful to the surgeons who contributed patients to this study. The work was supported by a grant from the Medical Research Council.

### References

BOOTH, S.N., JAMIESON, G.C., KING, J.P.G., LEONARD, J., OATES, G.D. & DYKES, P.W. (1974). Carcinoembryonic antigen in the management of colorectal carcinoma. *Br. Med. J.*, **4,** 183.

BUROKER, T., KIM, P.N., GROPPE, C. & 11 others. (1978). 5FU infusion with mitomycin-C versus 5FU infusion with methyl CCNU in the treatment of advanced colon cancer. *Cancer,* **42,** 1228.

DUKES, C.E. (1932). The classification of cancer of the rectum. *J. Pathol. Bacteriol.*, **35,** 323.

EGAN, M.L., LAUTENSCHLEGER, J.T., COLIGAN, J.E. & TODD, C.W. (1972). Radioimmunoassay of carcinoembryonic antigen. *Immunochemistry,* **9,** 289.

GOLD, P. & FREEDMAN, S.O. (1965*a*). Demonstration of tumor specific antigens in human colonic carcinomata by immunological tolerance and absorption techniques. *J. Exp. Med.*, **121,** 439.

GOLD, P. & FREEDMAN, S.O. (1965*b*). Specific carcinoembryonic antigens of the human digestive system. *J. Exp. Med.*, **122,** 467.

HERRERA, M., CHU, T.M. & HOLYOKE, E.D. (1976). Carcinoembryonic antigen (CEA) as a prognostic and monitoring test in clinically complete resection of colorectal carcinoma. *Ann. Surg.*, **183,** 5.

HINE, K.R. & DYKES, P.W. (1984). A prospective randomised trial of early cytotoxic therapy for recurrent colorectal cancer detected by serum CEA. *Gut,* (In press).

HINE, K.R., LEONARD, J.C., BOOTH, S.N. & DYKES, P.W. (1978). Carcinoembryonic antigen concentrations in undiagnosed patients. *Lancet,* **ii,** 1337.

LAURENCE, D.J.R., STEVENS, U., BETTELHEIM, R. & 6 others. (1972). Role of plasma carcinoembryonic antigen in diagnosis of gastrointestinal, mammary and bronchial carcinoma. *Br. Med. J.*, **iii,** 605.

MACH, J.-P., JAEGER, P.H., BERTHOLET, M.-M., RUEGSEGGER, C.-H., LOOSLI, R.M. & PETTAVEL, J. (1974). Detection of recurrence of large-bowel carcinoma by radioimmunoassay of circulating carcinoembryonic antigen (CEA). *Lancet,* **ii,** 535.

MOERTEL, C.G., SCHUTT, A.J., HAHN, R.G. & REITEMEIR, R.J. (1975). Therapy of advanced gastrointestinal cancer with a combination of 5FU, methyl CCNU and vincristina. *J. Natl Cancer Inst.*, **54,** 69.

MOERTEL, C.G., SCHUTT, A.J. & GO, V.L.W. (1978). Carcinoembryonic antigen test for recurrent colorectal carcinoma. Inadequacy for early detection. *J. Am. Med. Ass.*, **239,** 1065.

RITTGERS, R.A., STEELE, G., ZAMCHECK, N. & 7 others. (1978). Transient carcinoembryonic antigen (CEA) elevations following resection of colorectal cancer: A limitation in the use of serial CEA levels as an indicator for second-look surgery. *J. Natl Cancer Inst.*, **61,** 315.

SOROKIN, J.J., SUGARBAKER, P,H., ZAMCHECK, N., PISICK, M., KUPCHICK, H.Z. & MOORE, F.D. (1974). Serial carcinoembryonic antigen assays. Use in detection of cancer recurrence. *J. Am. Med. Ass.*, **228,** 49.

STABB, H.J., ANDERER, A., STUMPF, E. & FISCHER, R. (1978). Carcinoembryonic antigen follow-up and selection of patients for second-look operation in management of gastrointestinal carcinoma. *J. Surg. Oncol.*, **10,** 273.

SUGARBAKER, P.H., ZAMCHECK, N. & MOORE, F.D. (1976). Assessment of serial carcinoembryonic antigen (CEA) in post-operative detection of recurrent colorectal cancer. *Cancer,* **38,** 2310.

THOMPSON, D.M.P., KRUPEY, J., FREEDMAN, S.O. & GOLD, P. (1969). The radioimmunoassay of circulating carcinoembryonic antigen of the human digestive system. *Proc. Natl Acad. Sci.*, **64,** 161.

WATERHOUSE, J.A.H. (1974). *Cancer Handbook of Epidemiology and Prognosis.* Edinburgh: Churchill Livingstone, p. 119.

WOOD, C.B., BURT, R.W., RATCLIFFE, J.G., MALCOLM, A.J.H. & BLUMGART, L.H. (1978). Patterns of change in carcinoembryonic antigen (CEA) levels in patients developing recurrent colorectal cancer. *Gut,* **19,** A988.

WOOD, C.B., RATCLIFFE, J.G., BURT, R.W., MALCOLM, A.J.H. & BLUMGART, L.H. (1980). The clinical significance of the pattern of elevated serum carcinoembryonic antigen (CEA) levels in recurrent colorectal cancer. *Br. J. Surg.*, **67,** 46.